# Supplementary material for: Reduction and Growth Inhibition of Listeria monocytogenes by Use of Anti-Listerial Nisin, P100 Phages and Buffered Dry Vinegar Fermentates in Standard and Sodium-Reduced Cold-Smoked Salmon
Source: Foods. 2023 Dec 6;12(24):4391. doi: 10.3390/foods12244391 (PMC10743221; doi:10.3390/foods12244391)
Supplement: Supplementary file 1 [file foods-12-04391-s001.zip › foods-2745831-supplementary/Supplementary Figures_Tables/Table S1.pdf]

Table S1. Experiment 1: Anti-listerial effects on contaminated CS salmon treated with nisin, PGL or nisin + PGL during storage for 29 days at 8 °C.

**Treatment**[1, 2]	**Reductions (log) in *L. monocytogenes* levels during storage**[3]				
	**Day 1**	**Day 7**	**Day 12**	**Day 19**	**Day 29**
Nisin	0.5 (ns)[4]	0.9 (*)	0.9 (*)	1.0 (**)	0.7 (ns)
PGL	0.5 (ns)	1.1 (**)	1.1 (**)	1.4 (***)	1.4 (***)
Nisin + PGL	1.4 (***)	1.6 (***)	1.3 (***)	1.8 (***)	1.6 (***)

[1] See Materials & Methods and Table 1 for details of Experiment 1

[2]The CS salmon was treated with nisin (1 ppm), listeria phages (PGL, $5x10^7$ pfu/cm^2), or both

[3] The numbers represent the reductions (log) in *L. monocytogenes* levels obtained by the treatments compared to non-treated CS salmon on day 1, 7, 12, 19 and 29 after treatment. They were determined as the average reductions of the means per day and type of CS salmon.

[4] Significance levels: ns = nonsignificant (p>0.1); * (p=0.01-0.05); ** (p=0.001-0.01); *** (p≤0.001).
