# Supplementary material for: Reduction and Growth Inhibition of Listeria monocytogenes by Use of Anti-Listerial Nisin, P100 Phages and Buffered Dry Vinegar Fermentates in Standard and Sodium-Reduced Cold-Smoked Salmon
Source: Foods. 2023 Dec 6;12(24):4391. doi: 10.3390/foods12244391 (PMC10743221; doi:10.3390/foods12244391)
Supplement: Supplementary file 1 [file foods-12-04391-s001.zip › foods-2745831-supplementary/Supplementary Figures_Tables/Table S2.pdf]

Table S2. ANOVA data Experiment 1, 4 °C storage: ANOVA for analyses of effect of Verdad, anti-listerial treatments (nisin, PGL or both), days of storage and *L. monocytogenes* strain mix (8-strain vs. 10-strain)

	Df	Sum Sq	Mean Sq	F value	Pr(>F)	Explained variance	Significance levels[1]
Verdad	1	7	6.8	106.591	0.000	60.6	***
Treatment	3	27	9.1	142.285	0.000	23.9	***
Days	4	11	2.7	41.975	0.000	0.6	***
Mix	1	0	0.3	4.253	0.045	1.7	*
Verdad:Treatment	3	1	0.3	4.073	0.012	3.6	*
Verdad:Days	4	2	0.4	6.423	0.000	0.8	***
Treatment:Days	12	0	0.0	0.470	0.922	0.3	
Verdad:Mix	1	0	0.1	2.095	0.155	1.3	
Treatment:Mix	3	1	0.2	3.070	0.038	1.0	*
Days:Mix	4	0	0.1	1.776	0.151	6.1	
Residuals	43	3	0.1				

[1] Significance levels: none = nonsignificant (p>0.1); * (p=0.01-0.05); ** (p=0.001-0.01); *** (p≤0.001)
