# Supplementary material for: Reduction and Growth Inhibition of Listeria monocytogenes by Use of Anti-Listerial Nisin, P100 Phages and Buffered Dry Vinegar Fermentates in Standard and Sodium-Reduced Cold-Smoked Salmon
Source: Foods. 2023 Dec 6;12(24):4391. doi: 10.3390/foods12244391 (PMC10743221; doi:10.3390/foods12244391)
Supplement: Supplementary file 1 [file foods-12-04391-s001.zip › foods-2745831-supplementary/Supplementary Figures_Tables/Table S3.pdf]

Table S3 ANOVA data Experiment 1, 8°C storage: ANOVA for analyses of effect of Verdad, anti-listerial treatments (nisin, PGL or both), days of storage and *L. monocytogenes* strain mix (8-strain vs. 10-strain)

	Df	Sum Sq	Mean Sq	F value	Pr(>F)	Explained variance	Significance Levels[1]
Verdad	1	9	8.9	54.107	0.000	20.7	***
Treatment	3	24	7.9	47.466	0.000	66.4	***
Days	4	76	18.9	114.417	0.000	0.8	***
Mix	1	1	0.9	5.313	0.026	1.1	*
Verdad:Treatment	3	1	0.4	2.477	0.074	1.9	
Verdad:Days	4	2	0.6	3.339	0.018	1.3	*
Treatment:Days	12	1	0.1	0.738	0.707	0.1	
Verdad:Mix	1	0	0.1	0.447	0.507	1.3	
Treatment:Mix	3	2	0.5	3.098	0.037	0.4	*
Days:Mix	4	0	0.1	0.619	0.651	6.1	
Residuals	42	7	0.2			NA	

[1] Significance levels: none = nonsignificant (p>0.1); * (p=0.01-0.05); ** (p=0.001-0.01); *** (p≤0.001)
