# Supplementary material for: Reduction and Growth Inhibition of Listeria monocytogenes by Use of Anti-Listerial Nisin, P100 Phages and Buffered Dry Vinegar Fermentates in Standard and Sodium-Reduced Cold-Smoked Salmon
Source: Foods. 2023 Dec 6;12(24):4391. doi: 10.3390/foods12244391 (PMC10743221; doi:10.3390/foods12244391)
Supplement: Supplementary file 1 [file foods-12-04391-s001.zip › foods-2745831-supplementary/Supplementary Figures_Tables/Table S4.pdf]

Table S4 ANOVA data Experiment 2, 4 °C storage: ANOVA for analyses of effect of Provian-NDV (P-NDV), anti-listerial treatments (nisin, PGL or both), days of storage and type of salt (Standard (3 % NaCl) or sodium-reduced (2.1 % NaCl + 0.9% KCl).

	Df	Sum Sq	Mean Sq	F value	Pr(>F)	Explained variance	Significance Levels[1]
P-NDV	1	13	12.6	194.028	0.000	19.3	***
Treatment	3	14	4.8	74.009	0.000	64.6	***
Days	4	48	12.1	186.072	0.000	0.2	***
Salt	1	0	0.2	2.838	0.099	0.7	
P-NDV:Treatment	3	1	0.2	2.711	0.057	7.1	
P-NDV:Days	4	5	1.3	20.395	0.000	2.3	***
Treatment:Days	12	2	0.1	2.175	0.031	0.9	*
P-NDV:Salt	1	1	0.7	10.203	0.003	0.9	**
Treatment:Salt	3	1	0.2	3.283	0.030	0.3	*
Days:Salt	4	0	0.1	0.834	0.511	3.7	
Residuals	43	3	0.1				

[1] Significance levels: none = nonsignificant (p>0.1); * (p=0.01-0.05); ** (p=0.001-0.01); *** (p≤0.001)
