# Supplementary figures and images for: Reduction and Growth Inhibition of Listeria monocytogenes by Use of Anti-Listerial Nisin, P100 Phages and Buffered Dry Vinegar Fermentates in Standard and Sodium-Reduced Cold-Smoked Salmon
Source: Foods. 2023 Dec 6;12(24):4391. doi: 10.3390/foods12244391 (PMC10743221; doi:10.3390/foods12244391)

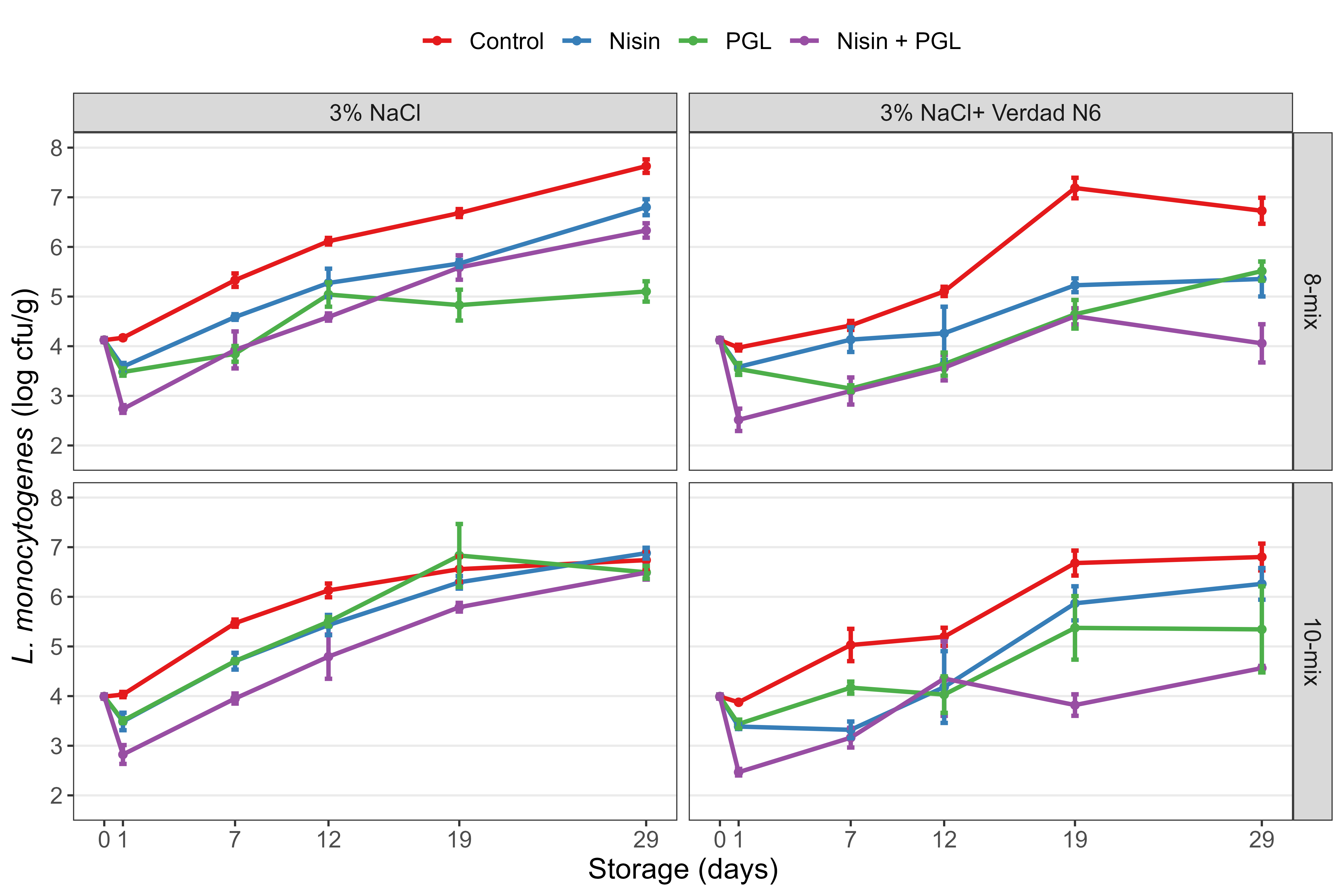

Supplement: Supplementary file 1 [file foods-12-04391-s001.zip › foods-2745831-supplementary/Supplementary Figures_Tables/Figure S1.tiff]
